# Coordinating collaborative infectious disease modeling projects with the hubverse

**DOI:** 10.1101/2025.10.03.25337284

**Published:** 2025-10-07

**Authors:** Consortium of Infectious Disease Modeling Hubs, Melissa Kerr, Rebecca Borchering, Alvaro Castro Rivadeneira, Lucie Contamin, Sebastian Funk, Harry Hochheiser, Emily Howerton, Anna Krystalli, Li Shandross, Nicholas G Reich

**Affiliations:** 1 University of Massachusetts Amherst, Amherst, MA, USA; 2 Influenza Division, National Center for Immunization and Respiratory Diseases, Centers for Disease Control and Prevention, Atlanta, GA, USA; 3 University of Pittsburgh, Pittsburgh, PA, USA; 4 London School of Hygiene and Tropical Medicine, London, UK; 5 Princeton University, Princeton, NJ, USA; 6 R-RSE SMPC

## Abstract

To better respond to a range of threats, decision-makers in diverse fields are increasingly interested in predictions they can understand and trust. Collaborative modeling can help increase the relevance, transparency, and robustness of predictions. This approach can be facilitated with hubs, or centralized data portals to collect, analyze, and communicate model output. This paper introduces the hubverse, an open-source suite of standards and software tools to streamline the creation and operation of collaborative modeling hubs. Hubverse file structure and model output standards enable the use of common tools to validate, aggregate, visualize, evaluate, and communicate model output. Currently, the hubverse is used by nearly two dozen collaborative and local modeling hubs around the globe to support infectious disease modeling efforts, including hubs hosted and/or used by the United States Centers for Disease Control and Prevention, the European Centre for Disease Prevention and Control, the Australia-Aotearoa Consortium for Epidemic Forecasting and Analytics, and the California Department of Public Health.

## Introduction

Reliable predictions can help decision-makers respond to threats as they unfold in many fields, including meteorology, economics, and public health. Predictive modeling is used to inform hurricane evacuations^1^ and central bank policy rate decisions^2^, and has also played a prominent role in initiating public health response to Ebola^3^ and informing COVID-19 restrictive measures^4^ and vaccination strategies^5^. As efforts to integrate predictive modeling into real-time decision-making increase^6,7^, effective communication between modelers and decision-makers is necessary to ensure that the latter have model output that they can understand, trust, and use.

The rise in infectious disease modeling efforts in recent decades has resulted in a complex landscape for decision-makers to navigate. This landscape has been characterized by a wide range of predicted outcomes with varying public health relevance and the use of diverse metrics to evaluate predictions^8^. These factors have made it difficult to compare models to determine which work best for a particular outcome, and, consequently, to select the best model to guide decisions^8^. During the early COVID-19 pandemic, a lack of coordinated modeling efforts hindered the utility of models to inform far-reaching policy decisions^9^. Yet even when modeling efforts are coordinated to address the same scientific question, different models often incorporate varying data sources, analytical methods, and assumptions about disease dynamics, potentially leading to conflicting results and recommendations^10,11^. Therefore, relying on any single model is problematic, as there is no guarantee that its underlying assumptions and methodologies will produce accurate or reliable predictions^10,11^. One effective solution is to use *ensemble* models, which aggregate predictions from multiple models. This approach allows for a more robust, comprehensive understanding of potential scenarios and helps mitigate the risks associated with model-specific biases or limitations^12^.

To facilitate ensemble building and improve the overall utility of modeling, collaborative modeling hubs can be established. We define collaborative modeling as a consortium of research groups that responds to a scientific challenge through coordinated modeling efforts^12^. We define a hub as a centralized data portal to collect, analyze, and communicate model predictions, i.e., *model output*. Collaborative modeling hubs offer many benefits. First, by imposing standards on model output, they make it easier to build ensemble models. Ensemble models have produced more accurate predictions than individual models in many situations^13^, including outbreak settings such as influenza^14^, COVID-19^15,16^, Ebola^17^, and dengue^18^. Since the advantages of ensembles have been routinely demonstrated, ensembles may be the primary hub product communicated^15,19,20^. Second, hubs may serve as a central communication point between modelers and stakeholders, helping to ensure that the outcomes being predicted and the model output generated meet decision-making needs^21^. Third, in eliciting model output submissions in response to a common challenge, hubs improve scientific rigor by encouraging model output transparency, data and methods sharing, and comparisons across models^12^. This builds understanding about which models or methods perform best, and can create a set of benchmarks for evaluation.

Numerous collaborative modeling efforts have been established over the past 10 years in response to different public health threats, including influenza, COVID-19, dengue, chikungunya, and Ebola^12^. One of the earliest collaborative modeling hubs for outbreaks was the FluSight influenza challenge, launched by the U.S. Centers for Disease Control and Prevention (U.S. CDC) in 2013 to forecast influenza trends 1–4 weeks into the future to improve risk assessment and preparedness. The FluSight challenge introduced quantitative forecasting standards and evaluation and cultivated a set of contributors from academia and industry^19^. When the COVID-19 pandemic hit, experience from FluSight helped inform the U.S. COVID-19 Forecast Hub. Launched in April 2020 in response to the massive amount of forecasts generated during the early pandemic, the U.S. COVID-19 Forecast Hub sought to standardize 1–4 week ahead forecasts of incident cases, deaths, and hospitalizations so that model output could be compared, aggregated into ensemble models, and communicated to stakeholders^22^. Other collaborative infectious disease modeling projects, not necessarily focused on prediction per se, have also attempted to address policy-relevant questions with multi-model approaches^5,23–25^.

The hubverse, the topic of the current paper, builds off the efforts of these, and other similar, early infectious disease forecasting hubs by introducing standards and tools to streamline collaborative modeling efforts and make it quicker and easier to set up hubs and communicate model output. Indeed, while collaborative modeling hubs can provide enormous value in better understanding threats, they can also be complex and time-consuming to set up. The hubverse introduces file structure and model output standards, which enable the use of tools to validate model submissions, aggregate, visualize, and evaluate model output, and communicate output through dashboards.

This paper offers an introduction to hubverse tools and standards. The Results section provides an overview of hubverse architecture, examples of hubs using hubverse tooling, and a detailed description of different user roles and how these users may interact with hubverse tools at the various phases of a hub. The Results section also presents a case study of the U.S. CDC’s FluSight hub, which began using hubverse infrastructure for the 2023–2024 influenza season to configure, accept, and analyze model output submissions. The paper then discusses the applications of hubverse-style modeling hubs and potential future directions. The Methods section details hub file storage structure and model output and data standards.

## Results

### Summary of hubverse architecture

We provide a brief summary of the architecture of a hubverse modeling hub, with details to be filled out over the course of the manuscript.

A hub is a data portal that contains data and metadata files that conform to specific file structures and data standards. Data are typically stored in a version-controlled repository, such as GitHub, though hubs can also mirror data to cloud storage services. Configuration files (JSON text files) in the hub configuration directory define how the hub is set up and the scientific challenge, or *modeling tasks*, to be addressed.

Hubs solicit model output submissions from modeling teams during specific time periods, or *rounds*, to address modeling tasks. For example, hubs may solicit model output once a week to predict weekly counts of hospital admissions due to influenza. Model output is submitted as individual files of tabular data to a hub’s model output directory, with one file representing one model and one round. Hubs may collect point predictions, which provide a single numerical estimate, or probabilistic predictions, which assign a likelihood to a range of outcomes. The latter are increasingly preferred by decision-makers and modelers in diverse fields since they provide a better representation of risk and uncertainty^26–28^.

For some predictions, predicted values of an outcome can be evaluated against actual observed values (e.g., forecasts of hospitalization trends can later be evaluated against actual observed hospitalizations). These actual observed values, referred to as *target data*, may be stored in a hub’s target data directory.

**Figure 1.**
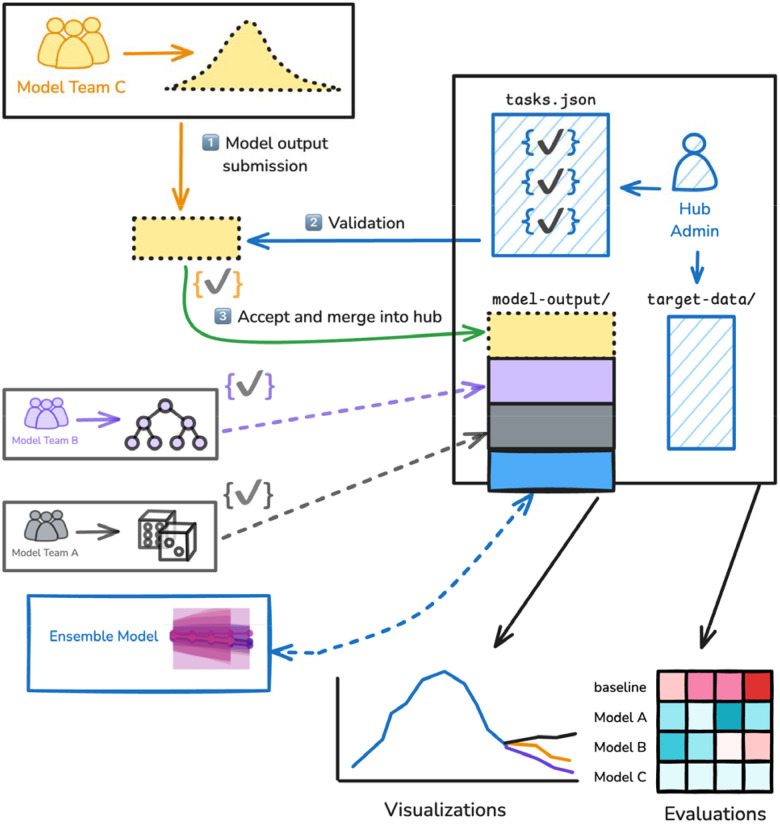
Overview of hub architecture. Modeling tasks are defined in the tasks.json configuration file. Model output submissions are collected in the model-output directory of a hub after being validated against the tasks.json configuration file. The optional target-data directory contains actual observed values of an event.

Hubverse data standards ensure that model output data can be seamlessly integrated to easily validate, aggregate, visualize, and evaluate predictions (Figure 1).

### Examples from the hubverse community

Hubverse infrastructure was developed by the Consortium of Infectious Disease Modeling Hubs, a team of software developers and researchers from academia with experience building and managing hubs who came together to streamline efforts following the COVID-19 pandemic. Upon its inception in 2022, hubverse infrastructure was initially adopted by groups that were part of the hubverse development team, though it has more recently been adopted by a number of groups both outside and inside academia that are not connected with the development team. Collaborative hubs using hubverse infrastructure have been set up around the globe to collect predictions from diverse modeling teams for a range of infectious diseases, including respiratory illnesses and arboviruses. Many of these hubs can be denoted as *community hubs* that have public repositories and are often open to any modeling team who would like to participate.

Hubverse infrastructure is also used by individual modelers or modeling teams to build *local hubs*, i.e., hubs that are set up on a laptop or cluster. Local hubs can be used to support local model development for general research purposes or to support future submissions to a community modeling hub. For example, a modeler could run and store iterations of model output in the local hub, and then use hubverse validation, visualization, and evaluation tools to analyze different models. While some local hubs may have public repositories, most will only ever exist on private local storage.

Table 1 shows all collaborative hubs that are known to the development team. For community hubs, we provide rows of model output data stored and hubverse-developed features used by the hub, including cloud storage and website dashboards. We also include some examples of local hubs with public repositories.

### Overview of user roles

**Figure. 2.**
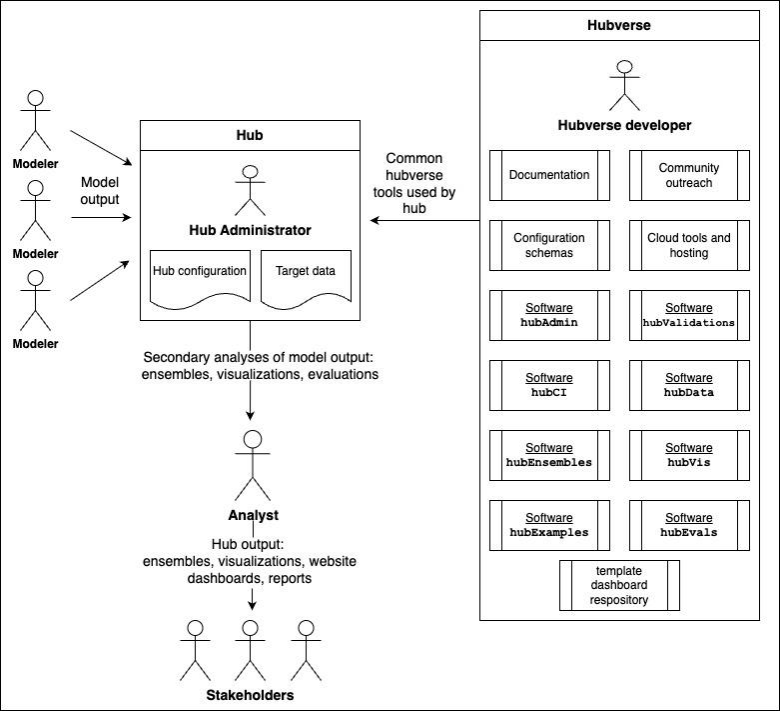
Schematic of the hubverse showing different user roles. The ecosystem of hubverse tools is shown in the box on the right. The ecosystem includes tools to set up and administer a hub, as well as tools to access, validate, aggregate, and visualize model output. A hub that follows the hubverse template is shown in the middle, with modelers contributing model output, hub administrators coordinating internal hub activities, and analysts processing model outputs into secondary products for stakeholders to consult.

The hubverse contributes to public health through the collaboration between users in different roles, of which we define five (Figure 2). We note that these are not mutually exclusive and a single person might serve in multiple roles, or a single role might be filled by multiple people:

- **Hub administrator**: the designer and maintainer of a hub. The hub administrator defines all details related to the scientific challenge to be addressed by the hub. Administrators set up the infrastructure to collect and validate model output submissions. If model output will be evaluated against target data, hub administrators ensure these data are available for use with hubverse evaluation tools.
- **Modeler**: the builder and maintainer of model code for a model whose outputs are submitted to the hub.
- **Analyst**: anyone who extracts data from the hub for secondary analyses, including model ensembles, visualization, and evaluation.
- **Stakeholder**: anyone who consults products derived from secondary data analyses of hub data, including local, state, and federal decision-makers or the general public.
- **Developer**: anyone who contributes ideas, code, or documentation to the hubverse ecosystem or who provides support to set up new hubs.

### How different users interact with hubverse tools at different phases of a hub

The lifecycle of a hub is organized around *rounds*, which we have previously defined as the specific time periods during which model output is solicited in response to a scientific challenge, or *modeling tasks*. The definition of a round may differ based on the hub. For example, hubs that accept daily submissions might consider each day a separate round, while other hubs may have a round every week, month, or season, with a submission period that may be open for multiple days. Based on this concept of a round, we define four phases in the lifecycle of a hub: 1) hub set-up, 2) active round, 3) post-round analysis, and 4) stakeholder products. Different user roles typically interact with the hub at different phases of its lifecycle. In the following subsections we describe the ways in which users interact with hubverse tools and products at different hub phases.

The hubverse landing page (https://hubverse.io) and documentation site (https://docs.hubverse.io/) provide access to hubverse data standards, tools, and documentation. Table 2 provides an overview of the hubverse tools that may be used by different users at each hub phase. **Supplementary file 1** provides a flowchart of the hub phases and the tools that are used in each phase.

#### Hub administrators

Hub administrators are involved in hub set-up and the day-to-day management of a hub. Hub administrators decide where to host and store hub data. The hubverse currently supports primary data storage on GitHub with an optional backup to Amazon Web Services Simple Storage Service (AWS S3). To set up a hub repository on GitHub, the hubverse website provides tutorials and template repositories (i.e., hubTemplate). Template repositories can be used to create a new repository that can be cloned to a computer to work with locally. Template repositories include the three required configuration files (admin.json, tasks.json, and model-metadata-schema.json) that administrators will need to customize for their hub.

The most substantial of these, the *tasks* configuration file (see **Supplementary file 2** for an example of the tasks configuration file as used for the FluSight hub), defines the modeling task to be addressed by a hub. To complete this configuration file, administrators will need to consider:

- the quantitative predictions to collect, or *modeling targets* (e.g., incident COVID-19 hospitalizations);
- the steps ahead being predicted, or *horizon*, (e.g., 1–4 weeks in the future);
- other variables to be used in modeling, or *modeling task identifiers* (e.g., location);
- when to open rounds;
- what estimates to collect to represent the modeling targets, or *output type* (e.g., mean, median, quantile, probability mass function, etc.).

The hubAdmin R package^29^ can help create the tasks.json config file and can validate all configuration files.

Once configuration files are set up, hub administrators can accept model output submissions from modeling teams. The hub repository should contain a README file detailing submission acceptance criteria, including relevant dates, outcomes to be predicted, and data formatting requirements. Hub administrators will create directories within the hub repository to collect both model output and *model metadata* (i.e., information about the team submitting the model and the model being submitted). Hub administrators should ensure that all model output and metadata submissions are *validated*, i.e., tested against the JSON configuration files to ensure the usability and integration of the output in downstream tools for data ingestion, ensembling, visualization, and evaluation. The hubValidations R package^30^ can be used to validate individual files or to set up ongoing validations of model output submitted to a hub repository through a pull request. Hubs can also configure customized validation functions within standard hubValidations workflows if desired.

Optionally, hub administrators may set up continuous integration through GitHub Actions to validate model submissions. Continuous integration involves automating the way code and data are validated prior to being merged into a shared hub repository. The hubverse currently provides several GitHub Action templates that can be used by GitHub-hosted hubs to validate model submissions and upload data to AWS S3 storage.

When predicted values of an outcome can be evaluated against actual observations, administrators should ensure that target data are available and accessible. Target data may need to be specifically formatted as *oracle output* data for use with hubverse evaluation tools (see Methods section for more on target data formats).

After a given modeling round, hub administrators may perform routine data processing and report generation. They may use hubverse R packages for model ensembles, visualization, and evaluation as discussed later in the data analyst section. To communicate model output, administrators may create a simple website to provide model output visualizations and/or evaluation reports using hubverse dashboarding tools.

#### Modelers

The active round phase of a hub revolves around modeling team submissions. Accordingly, modelers are the key players in this phase and are responsible for: 1) submitting *model output* data and 2) submitting *model metadata* once for each model submitted. These files should be submitted to the GitHub repository. Modelers should carefully read the submission instructions located in the README file in the hub repository. In particular, modelers will need to understand the different model output types that define the format in which quantitative predictions are structured and submitted to a hub (see Methods for more on model output). Both model output and model metadata can be validated before submission using the hubValidations R package.

#### Data analysts

Data analysts perform secondary analyses of model output data in the post-round analysis phase of a hub. Data analysts may use hubverse tools to download model output to aggregate model output into ensembles, produce different model output visualizations, generate evaluation reports, or conduct secondary research on the model outputs.

The hubData R package^31^ leverages functionality from Apache Arrow^32^, allowing users to connect to, extract, query, and analyze model output data efficiently from the hub data store. Apache Arrow is a multi-language toolbox that defines a columnar memory data format for flat and nested data, allowing data systems to efficiently store, process, and move data^32^. Data analysts can connect to a fully configured hub or to the model output directory of a hub that has not been fully configured. In establishing a connection to a configured hub, the hubData package, using the R arrow package, creates a queryable dataset of files stored locally or in the cloud (analysts may also use standard tools for reading model output data, though this is less efficient).

The hubEnsembles package^33,34^ provides functionality to aggregate the output from multiple models into an ensemble using several common methodologies, such as Vincent ensembles and linear opinion pools. The hubEnsembles package supports both weighted and unweighted ensembles using unequal, user-specified weights for models.

Analysts may also want to visualize and evaluate model output against target data. The hubVis R package^35^ contains a function to plot predictions that look at various time points in the future, along with optional target data. The hubExamples R package^36^ provides example model output and target data for an example forecast hub, and demonstrates how to join observed target values with model output in order to facilitate direct comparisons. The hubEvals R package^37^ contains a function to merge model output with target data and compute scores.

#### Stakeholders

Stakeholders may interact with a hub through the consumption of hub products. We define three products of a hub that stakeholders are most likely to consult:

1. Ensemble models: aggregation of the output from usually at least 3–4 different models;
2. Website dashboard: a simple website with custom information pages as well as interactive visualizations and/or evaluation of model output;
3. Reports: customized material based on model output data to share with different groups of stakeholders.

#### Developers

Developers offer overarching support that is not phase specific. Developers may help hub administrators set up new hubs and contribute new ideas, code, and documentation to the hubverse. Developers may work to implement new infrastructure (e.g., cloud storage architecture) and/or work on feature requests or bug fixes for existing hubverse software. The hubExamples package^36^ contains example model output of different output types that can be used to develop unit tests or document functions with code samples.

All hubverse packages and tools are released under the open-source MIT license. Anyone interested in contributing to the hubverse can use and test hubverse software and tools, file issues, and share code. All contributors should read and abide by the contribution guidelines and Code of Conduct detailed on the hubverse website.

### Case study: U.S. CDC FluSight exercise

We use the FluSight Forecast Hub as a case study to demonstrate hubverse setup and functionality. The FluSight challenge, run annually by the U.S. CDC since 2013 (with a break for the 2021–2022 season due to reduced influenza activity during the COVID-19 pandemic), has served as a centrally coordinated collaborative effort to monitor and predict short-term influenza activity in the U.S. at the national, regional, and state level (https://github.com/cdcepi/FluSight-forecast-hub/)^19^. These seasonal collaborative forecasting challenges have garnered participation from dozens of academic, industry, and governmental research teams. Typically, forecasts have been submitted once a week from October through May (the influenza season in the U.S.). Forecasts consisted of quantitative predictions of observed values from public health surveillance systems that are consistently reported across the country. The exact data source and prediction targets have changed over the duration of the project.

Starting in the 2023–2024 season, FluSight has used the hubverse ecosystem to configure and maintain submissions of short-term forecasts (https://github.com/cdcepi/FluSight-forecast-hub/releases/tag/v1.0.0). **Supplementary file 2** shows the tasks configuration file for the FluSight hub. In this season, teams could choose to submit forecasts for one of two modeling tasks:

TASK 1: Predicting the count of new weekly hospital admissions due to influenza

TASK 2: Predicting the category corresponding to the rate of change in hospital admissions (i.e., “large_decrease”, “decrease”, “stable”, “increase”, “large_increase”)

For both tasks, forecasts were accepted for 53 locations (50 states, the U.S. as a whole, Puerto Rico, and Washington D.C.) and were made at the weekly scale. Forecasts were due to be submitted every Wednesday during the season. Forecasts could be made for five different weekly prediction horizons (−1 through 3), where weeks were defined as Sunday through Saturday and corresponded to the definition of “MMWR weeks” used by the CDC^38^. A prediction made for a horizon of −1 referred to the prediction for the MMWR week that ended on the Saturday prior to the submission date (the previous week), and a prediction made for a horizon of 0 corresponded to the week ending on the Saturday after the Wednesday submission due date (the current week). Key properties of the two modeling tasks are summarized in Table 3.

The rate change target has five valid categories (“large_decrease”, “decrease”, “stable”, “increase”, “large_increase”) that can each be assigned a probability, where the five probabilities must sum to one. The precise mathematical definitions to resolve the category for a given location and week are described in the FluSight challenge guidelines (https://github.com/cdcepi/FluSight-forecast-hub/blob/v1.0.0/model-output/README.md#rate-trend-forecast-specifications). We note here only that the definitions are created such that weeks or locations with low counts where distinguishing between noise from increases and decreases can be difficult are resolved to the “stable” category.

Using hubverse tools, the data also have been mirrored to the cloud, so a copy of the data lives in a publicly-accessible AWS S3 bucket. This means that instead of needing to download a local copy of the full repository to access the data, specific portions of the data for this project can be directly accessed from the cloud using hubData tools.

A detailed code demonstration is provided in **Supplementary file 3**.

## Discussion

### Predictive modeling to support decision-making across disciplines

The hubverse fills an important gap by providing a set of tools that standardizes predictive model output and stakeholder communication products. This open-source project provides infrastructure to 1) set up collaborative modeling hubs and collect model output submissions, and 2) serve as a central communication point for stakeholders.

There are common considerations across different fields that leverage the utility of predictive modeling for stakeholders. In many fields, probabilistic predictions are aggregated into ensemble models. Ensemble models originated in weather forecasting^39^ but have become best practices in many other fields, where they have been shown to result in improved accuracy^13,40^ and reliability^41^ and to produce more useful model output for decision-making^42^. The availability of data visualization tools to convey model output and uncertainty to stakeholders, especially those who are not mathematical experts, is increasingly important in order to build trust^6^.

Model performance also heavily influences the utility of predictive modeling for decision-makers^43^. Different evaluation metrics exist for different model output types. The generation of evaluation reports can provide evidence of model performance that can be used by decision-makers to select or discard models for use in decision-making^44^.

The hubverse software suite can be used to generate stakeholder communication products, including ensembles, interactive online dashboards, and evaluation reports. It is important to note that although the hubverse was developed by scientists working in the field of infectious disease, the infrastructure developed to set up collaborative modeling hubs and generate stakeholder products is general enough to have a broader range of applications, and could be used by groups starting up collaborative modeling efforts in different fields.

### Predictive modeling for public health questions

In providing a standard infrastructure for running, synthesizing, analyzing, and reporting model output, the hubverse helps eliminate unnecessary duplication of effort in setting up code for data processing, evaluation, ensemble building, and reporting across different hubs. Usage of a standard infrastructure lowers technological barriers to setting up a new hub, which could allow for expansion to other pathogens and faster set up during emerging public health threats. For example, the COVID-19 Forecast Hub could likely have been set up even earlier in the pandemic if hubverse infrastructure was available at the time. Results of a recent Council of State and Territorial Epidemiologists survey sent out to the epidemiology workforce in the U.S. support the urgency of predictive modeling output during a pandemic: 45 out of 50 U.S. states and territories polled indicated that predictive modeling would be useful in their jurisdiction to inform decision-making during the next public health emergency^45^.

In the field of infectious disease, hubverse-style hubs have been used to generate projections that respond to different public health questions. Forecasts are quantitative predictions of future disease trends, and can help answer questions about what is likely to happen in the near-term future. Forecasts can help guide decisions on operational planning and the amount of intervention needed to control disease spread. Nowcasts provide predictions about the current state of an outbreak by adjusting for data lags from a data stream up until the current date, helping to answer questions about what is happening now. They provide information about the current situation and can help guide strategies to manage immediate needs. Both nowcasts and forecasts can be evaluated against target data. In contrast, scenario projections are predictions that answer “what if” questions about disease trends if certain assumptions are met (e.g., disease transmissibility, vaccine efficacy or uptake, interventions, emergence of new variants). In contrast to forecasts and nowcasts, it is difficult to evaluate scenario projections since the assumptions these predictions are based upon are unlikely to ever be completely met. However, scenario projections can be useful tools to evaluate possible long-term patterns of transmission or the effectiveness of interventions, and may thus offer information for longer-term strategic planning^12^. For example, predictions generated by the U.S. COVID-19 Scenario Modeling Hub helped support decisions to broaden the COVID-19 vaccination program to 5–11 year olds^46^, hasten the distribution of bivalent vaccines in response to variants circulating at the time^5^, and administer boosters to a larger portion of the population^47^. In addition to the generation of nowcasts, forecasts, and scenario projections, hubverse-style hubs can also be used to aggregate estimates of disease parameters (e.g., incubation period, transmission rate, waning of immunity rate). Parameter estimation may be useful on its own to better understand epidemiological characteristics that can guide prevention and control measures. Moreover, parameter estimation results could also be incorporated into models to improve model performance.

### Contribution to open science

In addition to producing stakeholder products, the hubverse is notable for its contribution to collaborative modeling and open science. Many diverse scientific fields use collaborative modeling to establish benchmarks for comparison, identify and resolve errors, increase model transparency, and build ensembles. The Intergovernmental Panel on Climate Change, for example, has prioritized model comparison projects in which modeling groups perform a set of standard exercises and then researchers from around the globe evaluate the model output^48^. In the field of ecology, open community forecasting challenges have helped facilitate comparison among models and have shed light on factors affecting forecasting skill^49^. Violence forecasting hubs have placed an emphasis on the public availability of all input data, methods, and results and on transparency through system documentation and the public release of replication material and source code^50^. Hubverse infrastructure similarly promotes model output transparency, and also facilitates the sharing of methods used to build models through model metadata or model abstract submissions. Studies evaluating infectious disease models have found that some models may perform best in certain conditions, but no class of model has consistently superior performance. Ensemble models, made possible with model output transparency, have often outperformed their component models^14,51,52^.

The success of the hubverse is intertwined with its use of open source development and its contributions to open science. The open source nature of hubverse infrastructure development has made the hubverse a collaborative effort between many academic and non-academic research groups, promoting transparency and knowledge transfer and reducing dependence on any one research group. Since it is open source, hubverse infrastructure and tools are open to a wide audience, from modelers using it for individual model development/iteration to groups looking to set up large-scale collaborative hubs. The hubverse contributes to open science through its efforts to make model output data findable, accessible, interoperable, and re-usable (FAIR). Research has shown that establishing data and metadata standards helps improve findability and usability^53^. Model output standards contribute to interoperability by facilitating data synthesis and collaborative modeling efforts, including the production of ensemble models^53^. Model output standards also facilitate the production of information and documentation needed to access, understand, and reuse hub data^53^.

### Limitations

One limitation of the hubverse is its lack of easy uptake in low-resource settings, especially since the burden of infectious disease may be as much as ten times higher in low and middle-income countries^54^ and these countries are particularly vulnerable to emerging and re-emerging infectious diseases^55^. Although the hubverse aims to provide open source and well-documented infrastructure for setting up hubs, some degree of complexity is inherent, which may result in high start-up costs for new hubs. The current infrastructure is also currently only available in English, though capacity has been built in to accommodate other languages. In addition, successful hubs are ones that solicit model output from multiple modeling teams, resulting in the submission of a diverse set of models. Research suggests that at least four different models are needed to produce an ensemble model with robust performance as compared to the baseline model^56^. Soliciting these models may be challenging in low-resource settings without sufficient expertise or funding to offer groups who could build and run models.

Another limitation with the current hubverse model output data format is that it is tied to a tabular data structure that can be inefficient for larger datasets. This can limit the complexity of the modeling scenarios and/or the set-up needed for the hub. Additionally, data-type specifications for the “value” column can be challenging to handle when some tasks have outputs that require strings and other tasks require numeric values. Furthermore, hubverse tools are mainly available in R and are dependent on use with specific file formats (CSV, Parquet). However, development of some Python tools is currently in progress. Additionally, all hubs must currently be set up on a version-controlled repository like GitHub, although fully cloud-based hubs are a possible future addition to the hubverse.

### Future directions and vision for hubverse

There are a number of new, large-scale features and projects on the hubverse roadmap:

- Allowing for the creation of cloud-based hubs that can accept submissions directly, without needing to be mirrored to a repository-based hub, as is currently the case. Cloud-based hubs would permit larger file sizes, allowing for the collection of more samples and modeling tasks, including a breakdown into smaller subpopulations.
- Containerizing models that can be run on cloud infrastructure. This would allow for a hub to come with a few standard models that could be run on data provided by hub administrators, with a standard output to interface with the hubverse.
- Creating “benchmark hubs” that can serve as research-ready repositories for machine learning researchers looking to test new methods and compare them with existing models. These might include creating an archival hubverse version of several of the large modeling hubs that were run prior to the existence of hubverse standards.

In all, we see the hubverse as a community-developed tool for the moment. In a world where more attention is being paid to predictive modeling, the hubverse serves as a tool that improves the scientific rigor behind real-time and retrospective predictive modeling challenges.

## Methods

### File storage architecture

Hubverse-style hubs maintain a file-based data storage architecture. Hubs should contain the directories, subdirectories, and files shown in Table 4. In brief, all hubs should have a documentation file (such as a README file) at the top level containing information on the hub structure. All hubs must also have a hub configuration directory containing all configuration files. Hubs will have separate directories for model output and model metadata submissions from modeling teams. Hubs that predict outcomes that may be eventually evaluated against target data may store these data within an optional target data directory. If any code and scripts are present in a hub repository, they should be stored within the source code directory, and never within the model output directory.

While other options for data storage exist, namely a database, the decision to organize hub data storage around files was based on several pragmatic considerations. First, files are the natural submission unit for hubs. Using the original files allows a hub to take advantage of existing version-control software with time-stamps for clear audit trails of when files were submitted, as well as setting up clear “per-file” continuous integration actions, such as data validation or transformation. Second, when stored in an Arrow-compliant file-based structure, data at the scale of most current hubs can be retrieved with similar speed as if it were in a lightweight database (the hubverse development team ran some benchmarking experiments comparing a DuckDB to a specific Arrow-based partition of the files). Third, keeping a file-structure-based storage system lowers barriers to entry for groups that want to set up a hub and leverage hubverse tools but don’t have the capacity or technical expertise to stand up a database backend.

### Model output standards

Modeling team submissions are known as model output. Model output must follow a tabular representation where each row represents a single prediction (or one aspect of a prediction, like a single quantile value, or a single sample) and each column provides additional information about the prediction. Model output may be submitted as CSV or Parquet. Parquet is a non-human-readable file format that stores data by column, resulting in more efficient data compression and faster data querying^57^. Though Parquet may be more complex to work with than CSV since additional software may need to be installed, the use of Parquet may help in getting around GitHub file size limits.

Model output columns can be divided into four groups: (1) the *model identifier* indicates what model has produced the prediction, (2) the *task identifiers (task IDs)* provide details about what is being predicted, (3) *output representation* specifies how the prediction is reported (e.g., mean, median, quantile), and (4) *value* provides the prediction. The latter three groups must correspond to how modeling tasks were defined in the tasks.json configuration file (see **Supplementary file 2** for example tasks configuration from the FluSight hub). Example model output displaying model identifier, task identifier, output representation, and value columns are shown in Table 5.

#### Task IDs:

Task IDs define the variables included in the model output and how they should be represented. Each unique row of model output data is defined by a combination of task ID values. The composition of the task ID variables and their accepted values are hub specific. Commonly used (and optional) task IDs are shown in Table 6, but additional task IDs may be specified depending on the needs of a hub. The hub can specify the structure, data type, and valid values of all task ID variables.

Hubs often use a task ID variable to define a submission round, which may be as simple as using origin_date or forecast_date.

#### Output representation:

This component defines how the prediction is represented in model output submissions. The output_type column defines the type of representation of the predictive distribution, while the output_type_id provides additional identifying information specific to the output type. These two columns can be thought of as providing output metadata about the predicted value.

The *value* column provides the prediction (Table 7)

### Target data

Target data are the actual observed values of what is being predicted in model submissions. Hubs that evaluate model predictions against target data values should ensure that target data are available and accessible. This can most commonly be achieved by providing code to access target data or by storing target data snapshots in the target data directory within the hub repository. Target data can be represented in two forms: *time-series* or *oracle output*.

The first format is *time-series* data. This is often the native or “raw” format for data. Each row of the dataset contains one unit of observation. For example, if the number of influenza cases per week is being reported for each of several states, the unit of observation would be a location and week. The columns consist of:

1. Task ID variables that uniquely define the unit of observation, with one column representing the date.
2. An “observation” column with the observed value.

The second format is *oracle output* data. Oracle output data is derived from the time series data and represents model output that would have been generated if the target data values had been known in advance, as if by an oracle. Oracle output follows a format that is similar to a hubverse model output file, with three main differences:

1. Predictions correspond to a distribution that places probability 1 on the observed target outcome.
2. Predictions are stored in a column named òracle_valuè rather than `valuè. The implications of this depend on the output_type (Table 8).
3. Generally, oracle output columns will be a subset of the columns of valid model output, with just those columns that are needed to correctly align oracle_values with the corresponding model output predicted values.

This structure allows a model output dataset to be joined with an oracle output dataset so that the model output `valuè can be compared and evaluated against the corresponding òracle_valuè. Having these data together in the same row can be helpful for evaluation and visualization.

Table 9 shows an example of specially formatted oracle output data, which can then be joined to model output data for visualization and evaluation purposes.

### Technology and data formats used in development

All hubverse code is developed in public, open source repositories on Github. Hubverse data may live on GitHub and/or on cloud servers, such as AWS S3 buckets. The R^58^ and Python^59^ languages are used to develop user-facing libraries. The Apache Arrow^32^ columnar data format is used when data are read in by users.

Datafiles may be stored in CSV or Parquet formats. JSON format textfiles are used for hub configuration files and JSON or YAML files are accepted for model metadata. Cloud storage for hubverse repositories are set up using infrastructure-as-code services such as Pulumi.

Continuous integration, such as used for real-time validation of submitted model output, is implemented via GitHub Actions.

## Supplemental files

1. Flowchart of hub phases
2. U.S. CDC FluSight modeling tasks configuration file
3. U.S. CDC FluSight case study vignette

## Figures and Tables

**Table 1.** List of modeling hubs that use hubverse data standards. For each hub, we list its name and the group that runs the hub. For community hubs, we provide the associated GitHub repository containing the hub’s data, a count of the number of rows of tabular model-output data stored in the hub (as of June 2025), the number of models that have submitted to the hub, and indicators of whether the hub uses hubverse-developed features such as a website dashboard or cloud storage.

Hub	Owner	Year(s)	Repository (https://github.com/)	Rows of model output data (as of June 2025)	# Models	Hubverse-dev eloped dashboard	Cloud storage
**Community hubs**							
COVID-19 Scenario Modeling Hub*	Scenario Modeling Hub Coordination Team	*	midas-network/covid19-scenario-modeling-hub	626,219,176	28		
RespiCompass	European Centre for Disease Prevention and Control (ECDC)	2024-	european-modelling-hubs/RespiCompass	213,562,080	14		
SARS-CoV-2 Variant Nowcast Hub	Reich Lab at UMass-Amherst	2024-	reichlab/variant-nowcast-hub	204,133,734	6	✓	✓
Flu Scenario Modeling Hub*	Scenario Modeling Hub Coordination Team	*	midas-network/flu-scenario-modeling-hub	150,013,626	19		
RSV Scenario Modeling Hub*	Scenario Modeling Hub Coordination Team	*	midas-network/rsv-scenario-modeling-hub	55,188,380	18		
FluSight archive	Hubverse	2015–2020	hubverse-org/flusight_hub_archive	29,364,474	99		✓
FluSight	U.S. Centers for Disease Control and Prevention (CDC)	2023-	cdcepi/FluSight-forecast-hub/	13,832,587	70	✓	✓
COVID-19 Scenario Modeling Hub - Research*	Scenario Modeling Hub Coordination Team	*	midas-network/covid19-smh-research/	3,185,180	7		
COVID-19 Forecast Hub	CDC	2024-	CDCgov/covid19-forecast-hub	1,993,088	19	✓	✓
RespiCast Syndromic Indicators	ECDC	2024-	european-modelling-hubs/RespiCast-Syndro micIndicators	1,591,830	23		
US RSV Forecast Hub	Johns Hopkins University Infectious Disease Dynamics Group	2023-	HopkinsIDD/rsv-forecast-hub	1,117,639	9		
RespiCast Influenza archive	ECDC	2023–2024	european-modelling-hubs/flu-forecast-hub_a rchive	407,938	16		
RespiCast COVID-19	ECDC	2024-	european-modelling-hubs/RespiCast-Covid19	358,931	13		
AI4Casting Hub Respiratory Virus	University of Guelph	2024-	ai4castinghub/rvdss-forecast	255,640	9		
RespiCast ARI archive	ECDC	2023–2024	european-modelling-hubs/ari-forecast-hub_a rchive	218,844	12		
AI4Casting Hub Hospital Bed Occupancy	University of Guelph	2024-	ai4castinghub/hospitalization-forecast	90,055	6		
West Nile Virus Forecast Hub	California Department of Public Health	2024-	cdphmodeling/wnvca-2024	61,061	25		
Flu Metrocast Hub	University of Texas-Austin and UMass-Amherst	2025-	reichlab/flu-metrocast	49,968	7	✓	✓
**Collaborative hubs with private respositories**							
North Carolina Department of Health and Human Services/Atlantic Coast Center for Infectious Disease Dynamics and Analytics (ACCIDDA) Forecasting Collaboration	ACCIDDA		Private	Private	Private		
Paraguay Respiratory Virus Forecast Hub	University of Georgia/US CDC		Private	Private	Private		
Australia-Aotearoa Forecasting Hub	Australia-Aotearoa Consortium for Epidemic Forecasting and Analytics		Private	Private	Private		
**Local hubs**							
Variant Nowcast Model Development Retrospectiv	vReich Lab at UMass-Amherst		https://github.com/reichlab/variant-nowcast-m	1,291,807,776	8		
Flusion- Retrospective Hub	Reich Lab at UMass-Amherst		https://github.com/reichlab/flusion/tree/main/re	1,376,251	9		
Flusion- Submissions Hub	Reich Lab at UMass-Amherst		https://github.com/reichlab/flusion/tree/main/s	765,440	8		

* Pre-existing hub transitioning to hubvserse standards and tools.

† Based on model output data contained in the hub repository as of Sept. 2025.

**Table 2.** Structured overview of how different users interact with hubverse tools or products at each hub phase

Phase	Tool	User		
Hub set-up		Admin	Modeler	Analyst
Hosting/storing hub data	GitHub repositories, AWS buckets	✓		
Setting up a hub repository	Website tutorial, hubTemplate repository	✓		
Creating configuration files	Website tutorial, hubAdmin	✓		
Validating configuration files	hubAdmin	✓		
Defining modeling tasks conceptually	Website offers terminology and guidance, hubs need to customize	✓		
Setting up continuous integration	hubCI, GitHub Action templates	✓		
**Active round**				
Storing target data snapshots	Hub repository (optional)	✓		
Submitting model output	Hub repository		✓	
Submitting model metadata	Hub repository		✓	
Validating model submissions	hubCI, hubValidations	✓	✓	
**Post-round analysis**				
Connecting to model output data	hubData			✓
Building an ensemble	hubEnsembles			✓
Visualizing data	hubVis, hubExamples		✓	✓
Scoring model output data	hubEvals		✓	✓
Building reports	hubVis, hubEvals	✓		✓
Building a website/dashboard	hubVis, hubEvals, hub template dashboard repository	✓		✓

**Table 3:** Information about the two modeling tasks for the U.S. FluSight challenge in 2023–2024. The “wk inc flu hosp” target represented the number of new laboratory-confirmed influenza incident hospital admissions in a given week. The units of this target are a patient count. The variable type was specified as “continuous” because, even though the target is an integer count of patients, the continuous specification allows models to represent prediction interval values as fractional values (a reasonable modeling choice) without data validation errors.

name	output type	variable type	units
wk inc flu hosp	quantile	continuous	patient count
wk flu hosp rate change	probability mass function (pmf)	ordinal	categories based on admission rate per 100k population

**Table 4.** Structure of a hub, including required and optional directories, subdirectories, and files

HUB STRUCTURE				
Component	Name, location, and format within a hub	Description	Provided by:	
Documentation			Hub	Modeler
Documentation file	e.g., README.md file, located in top level of hub and within each directory	File containing info about the hub structure and additional details about each of the directories	✓	
**Hub configuration**				
Hub configuration directory	/hub-config/	Folder storing configuration files	✓	
Hub admin configuration file	/hub-config/admin.json	Structured text file containing overall configuration settings for the hub	✓	
Hub modeling tasks configuration file	/hub-config/tasks.json	Structured text file that defines modeling tasks and therefore implicitly defines the assumed structure for any model submitted	✓	
Hub model metadata configuration file	/hub-config/model-metadata-schema.json	Structured text file that defines the expected format of model metadata files submitted by modeling teams	✓	
**Modeling team submissions**				
Model output directory	/model-output/	Folder to collect modeling team model submissions	✓	
Model output subdirectory	/model-output/team1-modela	Model-specific subdirectory for submissions from one modeling team.		✓
Model output file	/model-output/team1-modela/<round-id1> <model_id>.csv or .parquet	Round-specific model submission file		✓
**Model metadata**				
Model metadata directory	/model-metadata/	Folder to collect modeling team model metadata submissions	✓	
Model metadata file	/model-metadata/team1-modela.yml or yaml	Model-specific metadata submission file		✓
**Model abstracts (optional)**				
Model abstracts directory	/model-abstracts/	Folder to collect optional round-specific model metadata	✓	
Model abstract subdirectory	/model-abstracts/team1-modela/	Model-specific subdirectory for round-specific model metadata		✓
Model abstract submission file	/model-abstracts/team1-modela/ <round-id1>.md	Round-specific model metadata submission		✓
**Target data directory (optional)**				
Target data directory	/target-data/	Folder storing actual observed (i.e., target) values of an outcome, links to external open-access sources, and/or information on how model targets can be calculated from target data	✓	
Time series data	/target-data/time-series.csv or .parquet	File with observed counts or rates	✓	
Oracle output data	/target-data/oracle-output.csv or .parquet	File containing data dervied from time series data; represents the model output that would have been generated if the target data values were known ahead of time.	✓	
**Auxiliary data directory (optional)**				
Auxiliary data directory	/auxiliary-data/	Folder to store any additional data related to modeling efforts	✓	
**Source code directory (optional)**				
Source code directory	/src/	Folder storing code that is present within the hub repository	✓	

**Table 5.** Example model output displaying columns for model identifier, task identifiers, output representation, and value columns

Model output								
Model identifier	Task identifiers					Output representation		Value
model_id	reference_date	target	horizon	location	target_end_date	output_type	output_type_id	value
* **mean**								
Flusight-baseline	2022-11-19	wk inc flu hosp	0	25	2022-11-19	mean	NA	51.18476
Flusight-baseline	2022-11-19	wk inc flu hosp	1	25	2022-11-26	mean	NA	51.39129
Flusight-baseline	2022-11-19	wk inc flu hosp	2	25	2022-12-03	mean	NA	51.89889
Flusight-baseline	2022-11-19	wk inc flu hosp	3	25	2022-12-10	mean	NA	52.54409
* **median**								
Flusight-baseline	2022-11-19	wk inc flu hosp	0	25	2022-11-19	median	NA	51
Flusight-baseline	2022-11-19	wk inc flu hosp	1	25	2022-11-26	median	NA	51
Flusight-baseline	2022-11-19	wk inc flu hosp	2	25	2022-12-03	median	NA	51
Flusight-baseline	2022-11-19	wk inc flu hosp	3	25	2022-12-10	median	NA	51
* **quantile**								
Flusight-baseline	2022-11-19	wk inc flu hosp	0	25	2022-11-19	quantile	0.05	22
Flusight-baseline	2022-11-19	wk inc flu hosp	0	25	2022-11-19	quantile	0.1	31
Flusight-baseline	2022-11-19	wk inc flu hosp	0	25	2022-11-19	quantile	0.25	45
Flusight-baseline	2022-11-19	wk inc flu hosp	0	25	2022-11-19	quantile	0.5	51
* **cdf**								
Flusight-baseline	2022-11-19	wk flu hosp rate	0	25	2022-11-19	cdf	0.75	0.56795162
Flusight-baseline	2022-11-19	wk flu hosp rate	0	25	2022-11-19	cdf	1	0.89112016
Flusight-baseline	2022-11-20	wk flu hosp rate	0	25	2022-11-20	cdf	1.25	0.96509880
Flusight-baseline	2022-11-21	wk flu hosp rate	0	25	2022-11-21	cdf	1.5	0.98509810
* **pmf**								
Flusight-baseline	2022-11-19	wk flu hosp rate category	0	25	2022-11-19	pmf	low	0.9999997
Flusight-baseline	2022-11-19	wk flu hosp rate category	0	25	2022-11-19	pmf	moderate	0.0000003
Flusight-baseline	2022-11-19	wk flu hosp rate category	0	25	2022-11-19	pmf	high	0.0000000
Flusight-baseline	2022-11-19	wk flu hosp rate category	0	25	2022-11-19	pmf	very high	0.0000000
* **sample**								
Flusight-baseline	2022-11-19	wk inc flu hosp	0	25	2022-11-19	sample	2101	−2
Flusight-baseline	2022-11-19	wk inc flu hosp	0	25	2022-11-19	sample	2102	2
Flusight-baseline	2022-11-19	wk inc flu hosp	0	25	2022-11-19	sample	2103	52
Flusight-baseline	2022-11-19	wk inc flu hosp	0	25	2022-11-19	sample	2104	47

* Blue rows label the output type for readability only and are not part of a standard model output file

**Table 6.** Table showing common task identifiers and a description of each. These are examples only, taken from common use cases in infectious disease modeling (where, for example, predictions may be wanted for multiple locations and age groups). New or additional variable names and structures can be defined by a hub, or alternate definitions of the below task ID variables could be adopted. The hubverse publishes this list of common variable names to encourage adoption of similar practices.

Task identifier	Description
origin_date or reference_date	A temporal starting point for the prediction. It can be used to calculate a target_date via the formula target_date = origin_date + horizon * time_units_per_horizon (e.g., with weekly data, target_date is calculated as origin_date + horizon * 7 days)
forecast_date	Date on which a model is run to produce a forecast. Alternative to origin_date or reference_date above.
scenario_id	Unique identifier for a scenario, if multiple scenarios are present in the tasks
location	Unique identifier for a location
target	Unique identifier for the target
target_date or target_end_date	For short-term forecasts, specifies the date of occurrence of the outcome of interest (e.g., if models are requested to forecast the number of hospitalizations that will occur on 2022-07-15, the target_date is 2022-07-15
horizon	Difference between the target_date or origin_date in time units specified by the hub (e.g., days, weeks, or months)
age_group	Unique identifier for an age group

**Table 7.** Table showing output and value representations

Output representation		Value
output_type	output_type_id	value
mean	Null (not used for mean predictions)	Numeric: the mean of the predictive distribution
median	Null (not used for median predictions)	Numeric: the median of the predictive distribution
quantile	Numeric between 0.0 and 1.0: a probability level	Numeric: the quantile of the predictive distribution at the probability level specified by the output_type_id
cdf	String or numeric: a possible value of the target variable	Numeric between 0.0 and 1.0: the value of the cumulative distribution function of the predictive distribution at the value of the outcome variable specified by the output_type_id
pmf	String naming a possible category of a discrete outcome variable	Numeric between 0.0 and 1.0: the value of the probability mass function of the predictive distribution when evaluated at a specified level of a categorical outcome variable.
sample	String or integer sample index	Numeric: a random draw from the predictive distribution. This is the only representation that allows for a joint distribution across, for example, horizon or locations. For a joint distribution, the index is used to denote trajectories.

**Table 8.** Table showing the implications of output_type on oracle_value in oracle data

output_type	oracle_value	output_type and output_type ID columns required?
mean, median, quantile, sample	observed value of prediction target	no
pmf	1 when the output_type_id corresponds to the observed category (indicating a probability of 1 for that category) and 0 for other categories	yes
cdf	0 for output_type_id levels that are less than the observed value and 1 for any levels that are greater than or equal to the observed value, corresponding to the step function cdf of a probability distribution that places all its probability at the observed value	yes

**Table 9.** Example oracle output data, which can be joined to model output data for visualization and evaluation purposes. Note that oracle output data will contain the subset of task identifier columns needed to align model output and oracle output data.

Oracle output data					
Task identifiers			Output representation		Oracle value
location	target_end_date	target	output_type	output_type_id	oracle_value
* **mean**					
25	2022-11-19	wk inc flu hosp	mean	NA	79
* **median**					
25	2022-11-19	wk inc flu hosp	median	NA	79
* **quantile**					
25	2022-11-19	wk inc flu hosp	quantile	NA	79
* **pmf**					
25	2022-11-19	wk flu hosp rate category	pmf	low	1
25	2022-11-19	wk flu hosp rate category	pmf	moderate	0
25	2022-11-19	wk flu hosp rate category	pmf	high	0
25	2022-11-19	wk flu hosp rate category	pmf	very high	0
* **cdf**					
25	2022-11-19	wk flu hosp rate	cdf	0.75	0
25	2022-11-19	wk flu hosp rate	cdf	1	0
25	2022-11-19	wk flu hosp rate	cdf	1.25	1
25	2022-11-20	wk flu hosp rate	cdf	1.5	1
* **sample**					
25	2022-11-19	wk inc flu hosp	sample	NA	79

* Blue rows label the output type for readability only and are not part of a standard oracle output file

## Data Availability

Datasets showing example model output and oracle output data are available in the hubExamples R package, which provides example data for forecasting and scenario modeling hubs in the hubverse format, https://github.com/hubverse-org/hubExamples. U.S. CDC FluSight model output and target data datasets from the 2023–2024 flu season are available on GitHub: https://github.com/cdcepi/FluSight-forecast-hub/releases/tag/v1.0.0. Hubs with publicly available datasets and S3 buckets are available here: https://hubverse.io/community/hubs.html

## Code Availability

All code related to the hubverse project is publicly available under an MIT License.

Code to create and validate hub configuration files is available in the hubAdmin R package: https://github.com/hubverse-org/hubAdmin.

Code to validate model output and model metadata submissions is available in the hubValidations R package: https://github.com/hubverse-org/hubValidations.

Code to set up continuous integration is available in the hubCI R package: https://github.com/hubverse-org/hubCI.

Code to connect to hub data is available in the hubData R package: https://github.com/hubverse-org/hubData.

Code to aggregate model output into ensembles is available in the hubEnsembles R package: https://github.com/hubverse-org/hubEnsembles.

Code to visualize model output is available in the hubVis R package: https://github.com/hubverse-org/hubVis.

Code to join observed target data values with model output in order to facilitate direct comparisons is available in the hubExamples R package: https://github.com/hubverse-org/hubExamples.

Code to evaluate model output is available in the hubEvals R package: https://github.com/hubverse-org/hubEvals.

Code related to all packages listed above is also available in the hubverse R package: https://github.com/hubverse-org/hubverse.
